# Bladder Sensory Neurons as Regulators of Neuro-Immune-Urothelial Interactions: From Molecular Mechanisms to Targeted Therapy for Neurogenic Bladder and Interstitial Cystitis/Bladder Pain Syndrome

**DOI:** 10.3390/cells15181715

**Published:** 2026-09-21

**Authors:** Shuaixiang Zheng, Jiaxin Wang, Xinqi Liu, Jiaming Liu, Abudukeremu Aierken, Qing Ling

**Affiliations:** 1 Department of Urology, Tongji Hospital, Tongji Medical College, Huazhong University of Science and Technology, Wuhan 430030, China; zhengshuaixiang99@163.com (S.Z.); jiaxinbiscuit@163.com (J.W.);; 2 Hubei Provincial Clinical Research Center for Minimally Invasive Treatment of Urology, Wuhan 430030, China

**Keywords:** bladder sensory neurons, neuro-immune-urothelial axis, neurogenic bladder, interstitial cystitis/bladder pain syndrome, peripheral sensitization, neuromodulation

## Abstract

**Highlights:**

**What are the main findings?**

- Bladder sensory neurons coordinate urothelial and immune responses through neuropeptide, purinergic, neurotrophic, and ion-channel signaling.
- Disease-specific afferent plasticity links local bladder inflammation to spinal and central sensitization.

**What are the implications of the main findings?**

- Therapeutic targets should be selected according to disease phenotype, cellular source, target cell, and level of clinical evidence.
- Combining molecular interventions with neuromodulation may disrupt persistent neuro-immune-urothelial feedback loops.

**Abstract:**

Bladder sensory neurons are essential components of lower urinary tract sensory encoding and micturition reflex control. They detect bladder filling, stretch, inflammatory mediators, and pathogen-associated stimuli, and they communicate with urothelial cells, immune cells, and stromal cells through neuropeptides such as calcitonin gene-related peptide (CGRP), substance P (SP), and vasoactive intestinal peptide (VIP). Under physiological conditions, this neuro-immune-urothelial axis contributes to bladder homeostasis. Under pathological conditions, including neural injury, infection, inflammation, or epithelial barrier disruption, TRP channels, purinergic signaling, neurotrophic factors, chemokines, and voltage-gated ion channels can induce peripheral sensitization, increased excitability of dorsal root ganglion (DRG) neurons, spinal plasticity, and central sensitization, thereby contributing to urinary frequency, urgency, bladder pain, and detrusor overactivity in neurogenic bladder and interstitial cystitis/bladder pain syndrome (IC/BPS). This review summarizes the anatomical and functional organization of bladder sensory afferents, sensory neuron-associated molecular signaling, neuro-immune-urothelial interactions, disease-specific mechanisms, and targeted therapeutic strategies. We also discuss the translational potential and limitations of interventions targeting TRPV1/TRPV4/TRPA1, P2X3, CGRP, SP/neurokinin receptors, NGF, ion channels, botulinum toxin, and neuromodulation. These mechanisms provide a framework for understanding neurogenic mechanisms in bladder disorders and for developing targeted interventions.

## 1. Introduction

This is a mechanism-focused narrative review rather than a systematic review or meta-analysis. Peer-reviewed literature was identified through targeted PubMed searches and backward reference screening of relevant primary studies and reviews. Searches were updated through July 2026 and combined terms related to bladder afferents or sensory neurons, the urothelium, neuroimmune signaling, neurogenic bladder, spinal cord injury, IC/BPS, pain, and neuromodulation. Human randomized or interventional studies, human functional and biopsy data, and human ex vivo studies were prioritized; mechanistically informative animal and in vitro studies were used when human evidence was unavailable. Evidence was considered in descending order of clinical translatability: (1) human interventional or functional evidence; (2) human tissue association data; (3) human ex vivo evidence; (4) causal evidence from animal models; and (5) in vitro mechanistic evidence. No formal risk-of-bias assessment or quantitative synthesis was performed, and pathways lacking human validation are explicitly identified.

Bladder sensory neurons are fundamental to maintaining lower urinary tract homeostasis. They detect mechanical signals associated with bladder filling, local chemical cues, and pathogen-associated stimuli, and they participate in bladder function by modulating the micturition reflex and neuro-immune crosstalk [1]. Their abnormal activation or phenotypic remodeling is closely associated with the development and progression of neurogenic bladder (NB) and interstitial cystitis/bladder pain syndrome (IC/BPS) [2,3]. Increasing evidence indicates that bladder sensory neurons release neuropeptides, including tachykinins and CGRP, thereby forming a complex network of interactions with blood vessels, urothelial cells, and immune cells. This network is commonly conceptualized as a neuro-immune axis [4,5]. For example, CGRP released by ILC2 cells in the bladder can activate RAMP1-positive monocytes and exacerbate bladder inflammation, abnormal voiding behavior, and abdominal mechanical hypersensitivity [6]. In vitro, H2O2 activates TRPA1 channels in bladder tissue, triggering the release of SP and PGE2 from sensory nerve terminals and enhancing spontaneous bladder contractions [7]. In addition, urothelial cells act as key network integrators. They possess sensory-like functions; detect mechanical stretch, local chemical changes, and pathogen-associated stimuli; and translate luminal signals into biological cues recognizable by suburothelial sensory nerves and immune cells through ATP, nitric oxide, acetylcholine, and other mediators [8].

Based on these findings, this review discusses the dual roles of bladder sensory neurons in disease. On the one hand, they act as direct effector cells that drive bladder motor dysfunction and hypersensitivity through abnormal signal transduction. On the other hand, they function as regulatory hubs that amplify inflammatory cascades and compromise epithelial barrier integrity through neuro-immune pathways. By integrating the regulatory networks of tachykinins, CGRP, and other key mediators, this review evaluates sensory neuron-targeted interventions, including neurotransmitter/receptor agonists or antagonists and neurostimulation strategies, as potential approaches for mechanism-based treatment.

## 2. Anatomical Organization and Functional Features of Bladder Sensory Neurons

### 2.1. Anatomical Basis of Bladder Sensory Afferent Pathways

**Figure 1:**
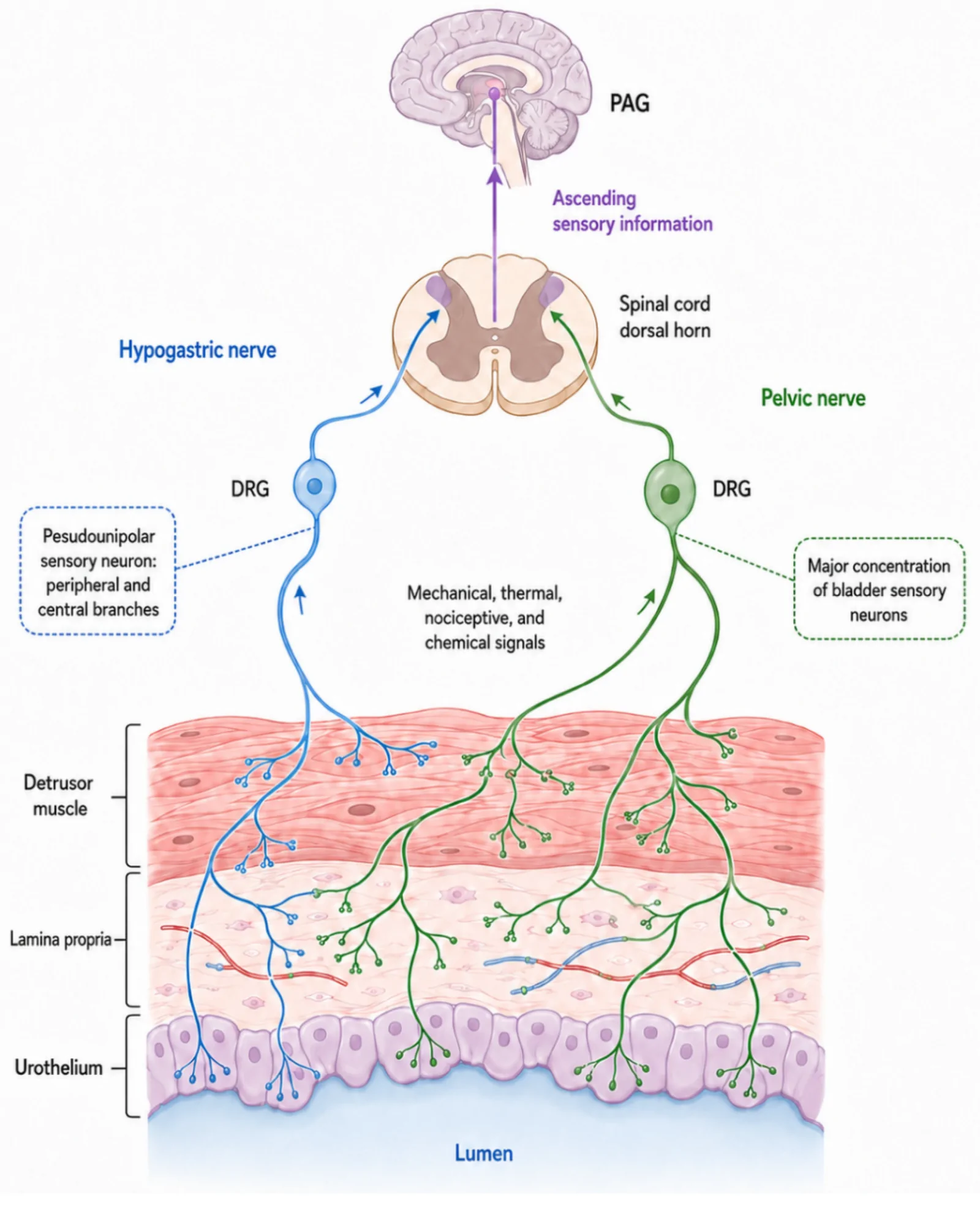
Simplified schematic of the anatomical distribution and afferent pathways of bladder sensory neurons. Bladder sensory information is mainly transmitted through the hypogastric and pelvic nerves to the corresponding dorsal root ganglia and is then projected to the spinal dorsal horn and supraspinal centers such as the periaqueductal gray. Peripheral terminals are distributed near the urothelium, lamina propria, and detrusor muscle and participate in the detection of mechanical, nociceptive, and chemical stimuli.

The cell bodies of bladder sensory neurons are mainly located in thoracolumbar (T10-L2) and lumbosacral (L5-S1) dorsal root ganglia, with a greater concentration in the latter [9]. DRG neurons are pseudounipolar neurons. Their peripheral branches innervate visceral, cutaneous, and musculoskeletal tissues, whereas their central branches enter the spinal cord via the dorsal roots and synapse with neurons in the dorsal horn, transmitting somatic and visceral information such as mechanical, thermal, nociceptive, and chemical signals [10,11]. Bladder afferent information is transmitted primarily through the hypogastric nerve, which corresponds to the thoracolumbar pathway, and the pelvic nerve, which corresponds to the lumbosacral pathway (Figure 1). These afferents enter the corresponding DRG segments and project to relevant regions of the spinal dorsal horn [12]. After entering the spinal cord, ascending sensory information is relayed through the periaqueductal gray (PAG), which integrates ascending excitatory input from the spinal cord and evaluates current bladder activity [13]. Peripherally, axonal terminals widely project to and around the urothelium, lamina propria, detrusor muscle, and perivascular regions, forming a complex sensory network [14,15].

### 2.2. Functional Features of Bladder Sensory Afferent Pathways

Bladder sensory neurons initiate lower urinary tract afferent signaling. Their main function is to detect bladder filling, wall tension, local chemical stimuli, and noxious stimuli, encode these peripheral signals, and transmit them to the central nervous system, thereby contributing to bladder sensation and micturition reflex regulation [1,16,17]. Functionally, bladder afferents are generally classified into myelinated A-delta fibers and unmyelinated C fibers [18]. A-delta fibers are mainly sensitive to physiological bladder filling and transmit volume-related mechanical signals, making them essential for normal storage and the initiation of micturition. C fibers are relatively quiescent under normal conditions but are highly responsive to inflammatory mediators, chemical stimuli, intense stretch, and tissue injury. They therefore play a prominent role in nociceptive transmission [19,20]. These afferent populations do not function in isolation but cooperate under physiological and pathological conditions to encode bladder sensation in a graded and finely regulated manner.

DRG neurons integrate mechanical and chemical signals through ion channels expressed on their terminals and on urothelial cells. Bladder afferent signaling can involve PIEZO2, TRPV4, ASIC3, P2X3, and other TRP-family channels. Rodent studies indicate that PIEZO2, TRPV4, and ASIC3 contribute to stretch- or acid-evoked bladder sensory transduction [21,22,23,24], whereas ATP-P2X3, TRPV1, and TRPA1 signaling is associated with inflammation-related afferent sensitization and bladder hyperreflexia [25,26]. Apart from early human trial evidence for intravesical capsaicin in neurogenic detrusor overactivity [27], these molecular mechanisms are supported predominantly by animal or ex vivo studies; their disease specificity and therapeutic implications are considered below.

## 3. Molecular Signaling Mechanisms Mediated by Sensory Neurons

**Figure 2:**
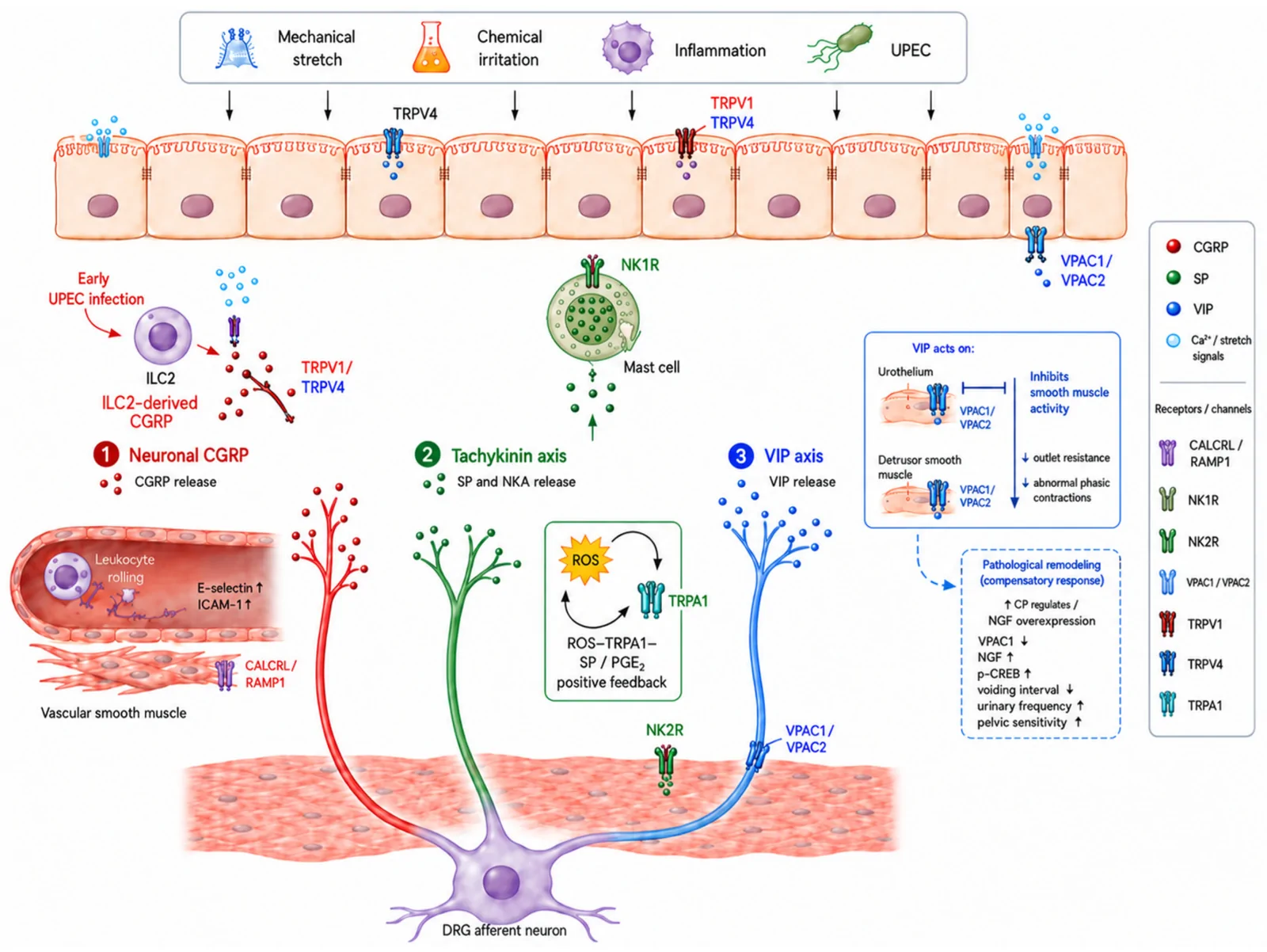
Bladder sensory neuropeptide networks and their roles in sensory signaling, inflammation, and motor regulation. Human tissue and ex vivo studies support the presence of TRPV1/CGRP-positive sensory fibers, SP-related neuroimmune associations, and VIP/VPAC-mediated modulation of bladder smooth muscle activity. The remaining components are supported by animal or experimental models.

Mechanical stretch or chemical irritation of the bladder can activate sensory neurons and induce the release of neurotransmitters and neuropeptides, including CGRP, SP, and VIP (Figure 2). These mediators dynamically regulate bladder motility and inflammatory responses and form a central signaling network for bladder homeostasis [28]. Over the past decade, immunostaining and multimodal studies of animal tissues have shown that bladder sensory afferents mainly release SP and CGRP as key neuropeptides involved in nociceptive signaling [29].

### 3.1. CGRP

In the bladder, CGRP is mainly released by capsaicin-sensitive C-fiber sensory neurons, whose terminals are concentrated in the trigone and submucosal layer [30]. Non-neuronal sources of CGRP are also present in the bladder. During early uropathogenic Escherichia coli (UPEC) infection, type 2 innate lymphoid cells can become a major local source of CGRP [6]. On the receptor side, the functional CGRP receptor, composed of CALCRL and RAMP1, is expressed not only on monocytes/macrophages but also on submucosal fibroblasts, PDGFR-alpha-positive interstitial cells, and vascular smooth muscle cells [31,32,33]. This spatial arrangement of ligand and receptor provides the structural basis for CGRP-mediated paracrine regulation in the bladder microenvironment.

Under pathological conditions, CGRP signaling may be aberrantly activated and can amplify bladder inflammation. For example, local application of capsaicin to the rat bladder rapidly induces leukocyte rolling, adhesion, and upregulation of E-selectin/ICAM-1, whereas local application of the CGRP receptor antagonist CGRP8-34 substantially reduces the microvascular inflammatory response [34]. In rats, blockade of CGRP signaling can reduce micturition frequency and pelvic pain and restore tight junction integrity between the urothelium and mucosa [31,35]. At the level of sensory transduction, CGRP provides a biochemical basis for ion-channel sensitization. Rodent studies indicate that upregulation of TRPV1 and TRPV4 in bladder sensory nerves and the urothelium is often accompanied by CGRP release. Together, these events are associated with reduced bladder capacity, detrusor excitation, mechanical hyperalgesia, urinary frequency, and pain [36,37]. Immunofluorescence of human cystitis tissue has shown colocalization of TRPV1 and CGRP in bladder sensory fibers [38]. This finding supports the presence of this pathway in human disease, whereas the animal models above further support a causal role for CGRP in inflammatory amplification; however, this causal relationship has not been directly validated in humans. CGRP signaling is also involved in central nociceptive processing. In mouse models of chronic pain, long-term potentiation in the spinal dorsal horn is accompanied by increased and structurally remodeled CGRP-positive sensory terminals, consistent with a contribution to central sensitization and chronic pain maintenance [39]. In rats, spinal perfusion of CGRP can induce dorsal horn neuronal sensitization and aggravate local symptoms [40]. In addition, CGRP receptor signaling in the central amygdala shows hemispheric lateralization in experimental bladder pain. Pharmacological inhibition of CGRP receptors in the right central amygdala attenuates bladder pain-like behavior, whereas inhibition in the left central amygdala does not produce the same analgesic effect, supporting a pronociceptive role of right-sided amygdala CGRP signaling [41]. Overall, CGRP remains predominantly a preclinical candidate pathway in IC/BPS and NB. No randomized human bladder pain trial has demonstrated therapeutic efficacy of a CGRP pathway antagonist, and direct human interventional validation is lacking. Therefore, CGRP should not be regarded as an established therapeutic target for bladder disease until corresponding clinical evidence becomes available.

### 3.2. Tachykinins

The tachykinin family, including SP, neurokinin A (NKA), and neurokinin B (NKB), and their receptors NK1R, NK2R, and NK3R play important and context-dependent roles in IC/BPS and NB. In the bladder, the tachykinin system shows a broad functional bias: the NKA-NK2R axis is more closely related to effector organ responses, particularly detrusor contraction [42], whereas the SP-NK1R axis is more involved in sensory afferent activity, inflammatory amplification, and bladder pain [43].

SP primarily promotes bladder afferent firing and enhances the micturition reflex via NK1R. During early acute inflammation, SP is rapidly released from sensory nerve terminals, representing an early step in neurogenic inflammatory cascades [44]. SP released into the peripheral microenvironment can directly drive bladder pain and urgency, even without overt histological injury and independently of the voiding reflex [45]. This pathological remodeling involves two major pathways. First, SP-driven sensitization depends strongly on immune cell participation. In bladder biopsies from patients with IC/BPS, increased SP-positive sensory nerve fibers are closely apposed to mast cells, suggesting that SP signaling may be associated with mast cell activation [46]. Second, tachykinin signaling is coupled to oxidative stress and TRP channels. The ROS-TRPA1-SP/PGE2 axis forms an important positive feedback loop: reactive oxygen species activate TRPA1 channels on sensory nerves, which promotes SP release, and SP may in turn enhance phasic contractions and bladder overactivity through NK2R-related pathways [7].

As disease becomes chronic, the pathological effects of the SP-NK1R axis extend beyond the bladder and involve central nociceptive circuits. In human IC bladder biopsies, increased NK1R mRNA expression suggests that SP-NK1R signaling may maintain local inflammation and sensory amplification [47]. At the central level, chronic bladder stimulation enhances NK1R and SP expression in the lumbosacral dorsal horn, especially at L6-S1, supporting a role in central sensitization and chronic pelvic pain [48]. Animal studies support this concept: in experimental autoimmune cystitis, the NK1R antagonist aprepitant reduces pelvic pain, improves voiding abnormalities, and alleviates bladder inflammation [49]. A multicenter, double-blind, randomized placebo-controlled trial found that the NK1R antagonist serlopitant reduced daily micturition frequency at selected doses in patients with overactive bladder [50]. Therefore, blockade of SP-NK1R signaling may offer a therapeutic strategy for bladder pain and dysfunction, but its clinical value remains to be established.

### 3.3. Vasoactive Intestinal Peptide

VIP is widely distributed in submucosal and detrusor nerve plexuses of the bladder wall. Through VPAC1 and VPAC2 receptors, it acts on the urothelium and detrusor and provides fine regulation among nerve terminals, epithelial cells, and local tissues [51,52]. In human and porcine ex vivo studies, VIP reduces tone and spontaneous contraction amplitude in the detrusor, trigone, and urethra [53]. In some rodent models, however, VIP does not show a marked mechanical contractile effect, suggesting that its actions may depend on species, tissue location, and experimental conditions [54].

During inflammation and dysfunction, VIP signaling undergoes compensatory remodeling. In cyclophosphamide-induced cystitis models, VIP and its receptors show tissue-specific dynamic changes [55]. In NGF-driven bladder hypersensitivity and dysfunction, pathology may not simply result from reduced VIP levels but may be associated with receptor-side imbalance, such as downregulation of VPAC1 in the urothelium and detrusor [56]. Genetic models also suggest an endogenous protective role for VIP. VIP knockout mice show shortened intervoid intervals, increased frequency, and enhanced pelvic/somatic sensitivity at baseline, and these abnormalities become more prominent after CYP-induced cystitis [57]. However, such knockout evidence is limited in both the number of studies and model diversity and is insufficient to demonstrate an endogenous protective role for VIP in human bladder disease. Animal studies further show that VIP deficiency is also accompanied by increased bladder NGF levels, enhanced p-CREB expression in lumbosacral DRG, and structural changes in the bladder wall, indicating that VIP limits bladder afferent hyperexcitability and pain sensitization through neurotrophic and sensory plasticity mechanisms [58]. These findings suggest that VIP may contribute to sensory and immune homeostasis, but the evidence is derived mainly from animal and ex vivo experiments, and direct human interventional studies targeting VIP/VPAC signaling are lacking. Its specific role and therapeutic value in human bladder disease require further validation in human tissue and functional studies.

## 4. The Neuro-Immune-Urothelial Axis

**Figure 3:**
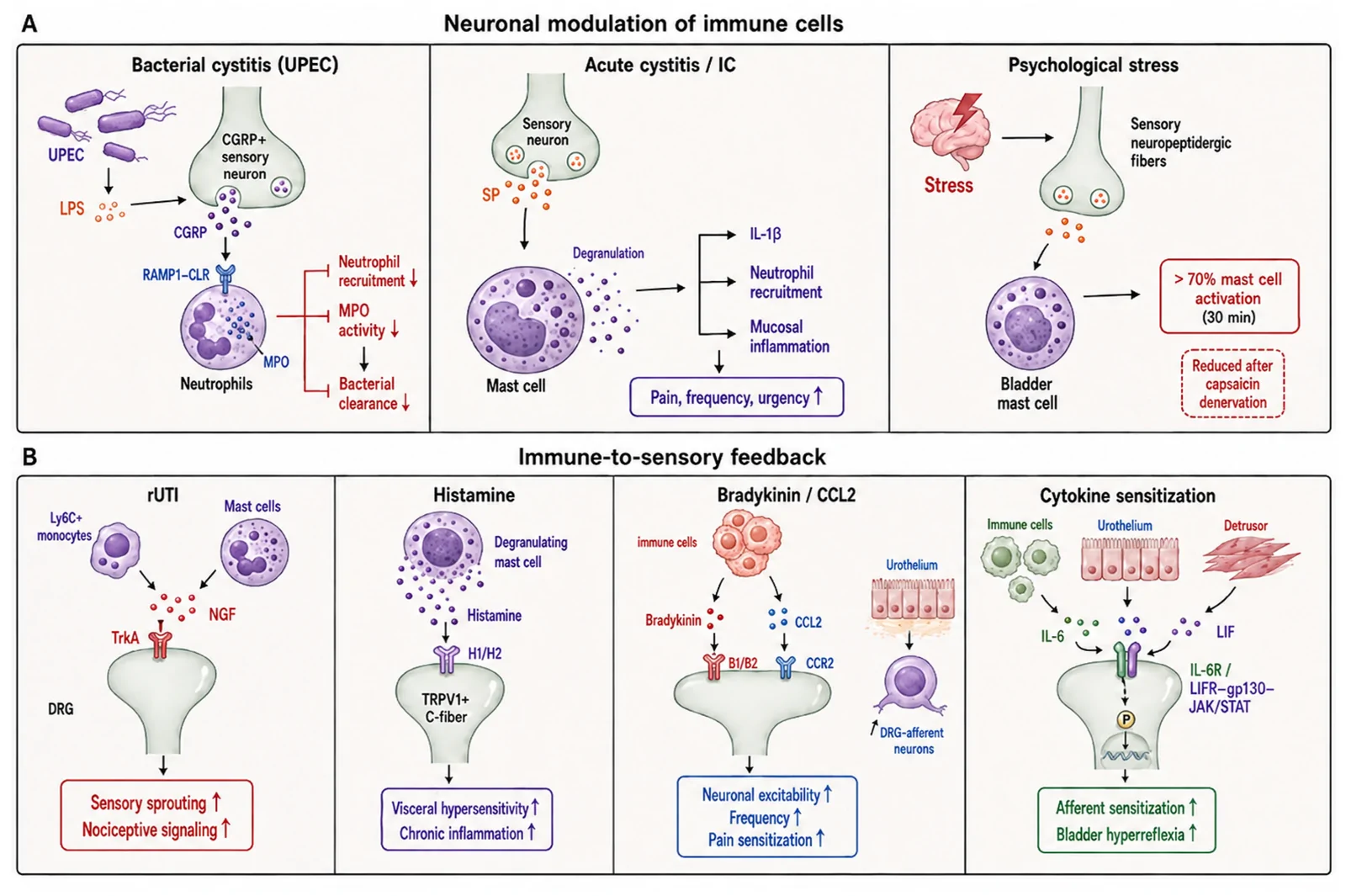
Bladder neuro-immune interaction model. Sensory neurons release neuropeptides such as CGRP and SP to regulate neutrophils, monocytes/macrophages, and mast cells. Conversely, immune cell-derived NGF, histamine, bradykinin, CCL2, and cytokines act on sensory neurons, promoting bladder afferent sensitization and inflammatory amplification. All directional regulatory arrows shown are based mainly on animal or mechanistic studies. Human studies currently provide primarily correlational observations, including changes in urinary SP and the spatial proximity of SP-positive sensory fibers to mast cells.

Bladder sensory neurons are key integrators in the neuro-immune-urothelial axis because they detect chemical and mechanical stimuli. This bidirectional signaling network regulates local immunity, epithelial barrier function, and inflammatory responses. Dysfunction of peripheral nerves or immune responses can disrupt the urothelial barrier, induce chronic inflammation, and promote the progression of bladder disease (Figure 3).

### 4.1. Neuro-Immune Interactions

Animal studies indicate that sensory neurons regulate immune cells by releasing neuropeptides. In UPEC-induced bacterial cystitis, LPS released by UPEC can directly activate bladder sensory neurons and promote CGRP release. Through the RAMP1-CLR axis, CGRP suppresses neutrophil recruitment and MPO activity, thereby reducing bacterial clearance [59]. In acute cystitis in rats, SP released by sensory nerves activates mast cell degranulation through the SP-NK1R axis; promotes IL-1beta-related inflammatory signaling, neutrophil recruitment, and mucosal inflammation; and aggravates pain, frequency, and urgency [60]. Human evidence is currently more often correlational at the urinary or tissue level; for example, increased urinary SP in patients with acute cystitis suggests that this axis may participate in mucosal inflammation and pain [61], but this does not establish causality. Such bladder neuro-immune crosstalk is also influenced by physiological and psychological stress. In animal studies, psychological stress activates bladder mast cells and worsens sensory and voiding abnormalities; these effects are attenuated after capsaicin-mediated depletion of many peptidergic sensory fibers, suggesting partial dependence on sensory neuron-derived neuropeptides [62,63].

Causal evidence that the immune and inflammatory microenvironment feeds back to enhance sensory afferent activity also comes mainly from model studies. Animal studies indicate that the bladder immune system also feeds back to sensory neurons through neural mediators. In recurrent urinary tract infection models, infiltrating Ly6C-positive monocytes and tissue-resident mast cells produce NGF, which acts on TrkA-expressing bladder-innervating DRG neurons. This drives abnormal sprouting of sensory fibers within the bladder wall and enhances nociceptive transmission [64]. In models of neurogenic cystitis and IC/BPS, histamine released by degranulating mast cells in the lamina propria acts directly on TRPV1-positive C fibers through H1/H2 receptors, sustaining visceral hypersensitivity and chronic inflammation [65]. Bradykinin from mast cells can also act through B1/B2 receptors on bladder afferents to enhance neuronal excitability and promote urinary frequency and pain sensitization [66]. In addition, CCL2 and CCR2 are upregulated in the bladder epithelium and bladder-related DRG afferents during inflammation and contribute to CYP-induced urinary frequency and pain sensitization [67]. Inflammatory cytokines such as IL-6 and LIF may also participate in afferent sensitization and bladder hyperreflexia via IL-6R/LIFR-gp130-JAK/STAT signaling. These factors arise not only from immune cells but also from the urothelium and detrusor [68]. Taken together, findings from these animal models suggest that immune-derived signals within the inflammatory bladder microenvironment can promote sensory afferent sensitization through multiple receptor-mediated and intracellular signaling pathways.

### 4.2. Urothelial-Neural-Immune Crosstalk

Current directional mechanistic evidence for urothelium–sensory nerve–immune crosstalk comes mainly from animal and experimental models. These studies suggest that the urothelium is no longer viewed as a passive physical barrier but functions as a sensory, secretory, and innate immune interface that detects mechanical stretch, chemical irritation, and pathogens and releases ATP, cytokines, and neurotrophic factors to maintain local homeostasis and drive pathological remodeling [69,70,71]. In physiological bladder-filling models, bladder filling induces ATP release, which activates P2X2/P2X3 receptors on suburothelial afferent terminals and encodes bladder sensation and the micturition reflex [72]. Urothelial TRPV4 channels regulate Ca^2+^ influx and ATP release, further amplifying primary afferent activity [73], providing experimental evidence that the urothelium participates in bladder mechanochemical sensory transduction. In a mouse model of acute bacterial cystitis, umbrella cells recognize luminal bacterial LPS through TLR4, release ATP, activate P2X2/P2X3-related afferent pathways, and enhance the micturition reflex. This flushing response contributes to early host defense before neutrophil-mediated bacterial clearance is complete [74]. Infection can also upregulate SP/NK1R signaling in neurons and the urothelium, generating a neuroepithelial activation loop that amplifies mucosal inflammation and pain; NK1R antagonists can attenuate inflammation, indicating that the urothelium participates not only in pathogen recognition but also in neurogenic inflammatory amplification [60].

**Figure 4:**
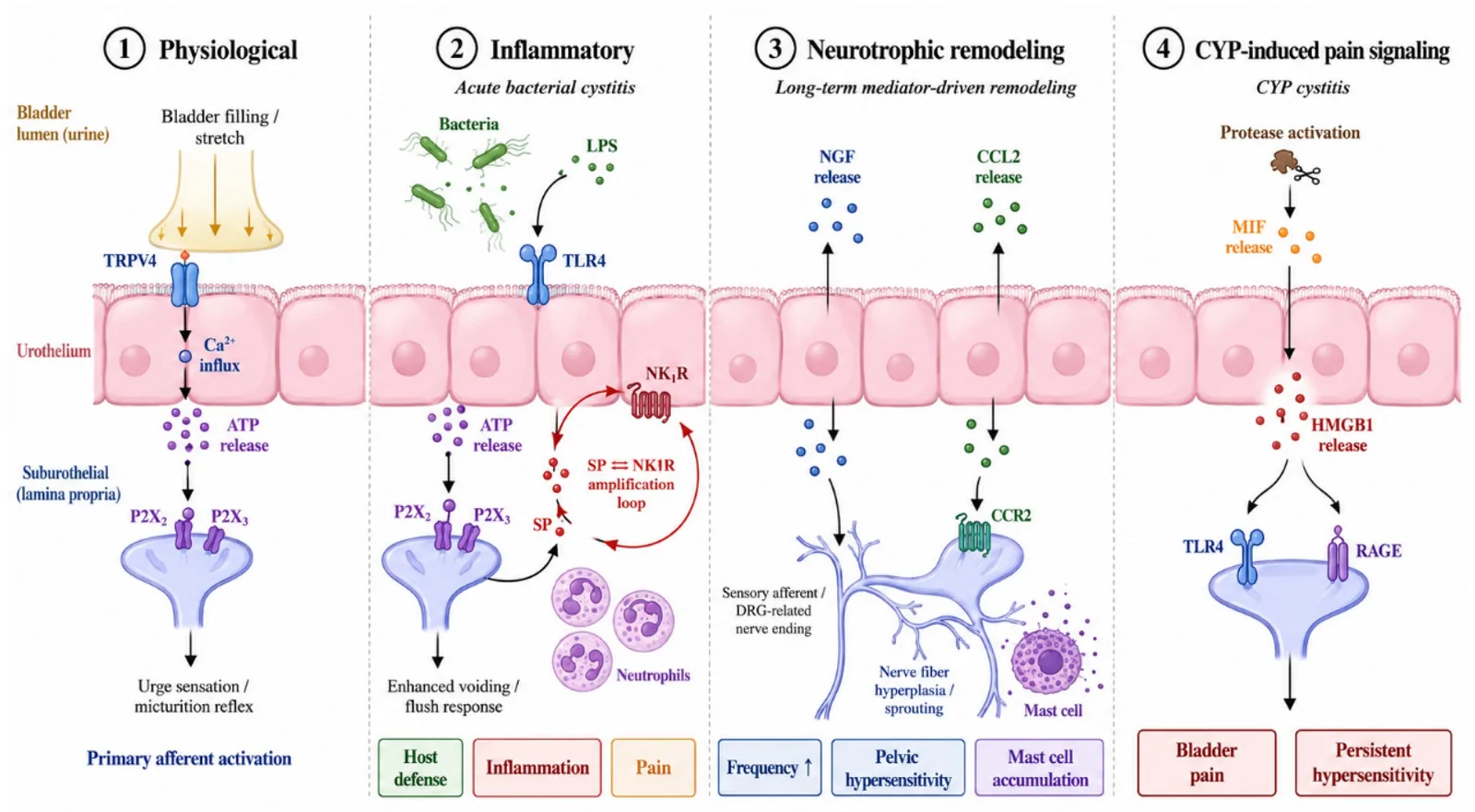
Roles of urothelium–sensory nerve–immune cell crosstalk in bladder homeostasis and disease. The urothelium detects stretch, pathogens, and inflammatory stimuli through receptors and channels such as TRPV4 and TLR4. It releases ATP, chemokines, NGF, MIF/HMGB1, and other mediators, thereby activating sensory afferents and immune responses and promoting urinary frequency, pain, and bladder hypersensitivity. The four groups of directional mechanisms shown are based mainly on animal or experimental models.

In models of chronic inflammation and neural remodeling, urothelial neurotrophic and inflammatory mediators can remodel the bladder neuroimmune microenvironment for prolonged periods. Urothelial-specific NGF overexpression induces nerve fiber proliferation in the submucosa and detrusor, increases voiding frequency and pelvic sensitivity, and is accompanied by focal mast cell accumulation, supporting the involvement of epithelial-derived NGF in both neuronal and immune remodeling in these models [75]. In CYP-induced cystitis in rats, CCL2/CCR2 is markedly upregulated in the urothelium and bladder-related DRG neurons; local CCR2 blockade increases bladder capacity and reduces frequency and somatic sensitivity, suggesting that epithelial chemokines may participate in afferent sensitization [76]. Protease activation can induce urothelial MIF release and promote HMGB1 export; extracellular HMGB1 then mediates bladder pain and persistent hypersensitivity through TLR4/RAGE-related pathways [77]. Disruption of urothelial tight junction integrity can also increase bladder afferent sensitivity in experimental models [78]. Overall, these animal and mechanistic studies support the urothelium as an important regulatory interface at which sensory and immune signals converge and provide relatively strong preclinical causal support for urothelium–sensory nerve–immune crosstalk [79] (Figure 4). However, sufficient human functional or interventional studies are lacking to show that these specific molecular pathways operate causally in human NB or IC/BPS in the same manner. Thus, this network is better regarded as a biologically plausible mechanistic framework and candidate direction for intervention rather than an established human disease pathway or therapeutic target.

### 4.3. Peripheral–Central and Cross-Organ Sensitization

Animal and anatomical studies suggest that urothelial ATP and other signals may participate in local sensory–immune interactions and may promote cross-organ sensitization through shared peripheral sensory pathways. Anatomical tracing has identified dual-projecting neurons in lumbosacral DRG that innervate both the bladder and colon, providing a direct substrate for pathological organ crosstalk [80]. Clinically, IC/BPS often coexists with irritable bowel syndrome and inflammatory bowel disease. Experimentally, colitis can induce bladder hypersensitivity, frequency, and detrusor instability, whereas cystitis can enhance colorectal afferent sensitivity, indicating bidirectional neural coupling [81]. In animal models, TNBS-induced colitis promotes bladder overactivity through CXCR4-ERK signaling in the DRG and spinal dorsal horn [82]. Colitis models also show increased CGRP, TRPV1, and related plasticity signals in bladder-associated DRG and spinal pathways, together with bladder hypersensitivity and voiding abnormalities [83]. These findings support a mechanistic basis for bladder–colon cross-sensitization, but they are derived predominantly from animal studies and do not establish that the same pathway drives disease in every patient with comorbid IC/BPS and bowel symptoms.

When abnormal peripheral input persists, pathological effects converge on second-order spinal neurons and drive central sensitization. Abnormal sensory information ascends through bladder–brain pathways to the PAG, thalamus, insula, anterior cingulate cortex, and supplementary motor area, which are also involved in normal bladder sensation and micturition control [84]. Neuroimaging studies show that patients with OAB have stronger activation of the insula and anterior cingulate during urgency [85]. In IC/BPS/UCPPS, fMRI and MAPP network studies show decoupling between the default mode network and the posterior cingulate/precuneus; enhanced connectivity between the posterior cingulate and pain/emotion-related regions such as the insula, thalamus, and amygdala; and reduced connectivity between the precuneus and regions such as the anterior cingulate and ventromedial prefrontal cortex [86,87]. These changes correlate with pain intensity, urinary symptoms, anxiety, depression, and reduced quality of life. ACC metabolites and ACC–limbic connectivity have also been associated with negative affect [88], and brain network measures may correlate with circulating inflammatory markers and anxiety or depression scores [89]. Longitudinal diffusion tensor imaging from the MAPP Network has further identified reproducible white matter microstructural abnormalities, some of which are associated with disease status or symptom severity [90,91].

From a translational perspective, central sensitization and cross-organ features are better suited as variables for patient stratification than as established therapeutic targets. Future IC/BPS clinical trials should prespecify directly clinically meaningful primary endpoints, such as bladder pain intensity, together with urgency, 24 h voiding frequency, or validated symptom scores to assess storage symptoms; neuroimaging, widespread pain sensitivity, and inflammatory/neuroimmune biomarkers can serve as secondary or exploratory mechanistic endpoints. Patients can be prospectively stratified according to bladder-localized versus widespread pain phenotypes, the presence of IBS or other chronic pain syndromes, psychosocial burden, pelvic floor function, and baseline widespread pain sensitivity. Intervention designs may compare bladder-targeted therapy alone with a dual-target strategy combining bladder treatment with central/neuromodulatory therapy; in patients with clear bowel comorbidity, bladder-only treatment may also be compared with combined bladder–gut intervention. Such stratified and controlled designs may help determine whether central and cross-organ abnormalities are accompanying disease markers or clinically relevant mechanisms that predict benefit from combination therapy.

## 5. Pathological Mechanisms of Sensory Neurons in Bladder Disorders

### 5.1. Neurogenic Bladder

Neurogenic bladder is a lower urinary tract storage and voiding dysfunction caused by central or peripheral nervous system injury. Among NB models, post-spinal cord injury bladder dysfunction has been most extensively studied in relation to sensory neuron remodeling [92]. After SCI, the bladder first undergoes transient areflexia. Subsequently, unmyelinated C fibers that are relatively silent under normal conditions become hyperexcitable and undergo axonal sprouting; they can replace A-delta fibers as a dominant source of afferent input for reflex voiding, forming a pathological spinal micturition reflex [93].

In the normal bladder, the micturition reflex mainly depends on A-delta afferents sensitive to bladder filling. After supraspinal spinal cord injury, loss of descending control unmasks capsaicin-sensitive C-fiber pathways, which acquire abnormal mechanosensitivity and become major drivers of neurogenic detrusor overactivity. Clinically, the ice-water test can evoke reflex voiding in patients with SCI and is considered a classic sign of C-fiber-mediated reflex activation [17]. In rats, removal or inhibition of C fibers reduces SCI-induced detrusor hyperreflexia [94]. After SCI, TRPV1-related bladder afferent pathways undergo structural and functional remodeling. In human NB bladder biopsies, TRPV1 immunoreactivity increases in the basal urothelium and TRPV1-positive nerve fiber density increases in the bladder wall [95]. In animal models, TRPV1-positive bladder afferent neurons show terminal axonal sprouting in the spinal cord and contribute to bladder hyperreflexia [96]. Collectively, these human tissue and animal findings support a shift from physiological afferent encoding to a pathological C-fiber-dominated reflex in SCI-related NB [97].

Bladder-innervating DRG neurons also show electrophysiological sensitization after SCI. Changes include somatic hypertrophy, lower action potential thresholds, increased Na+ currents, and reduced A-type K+ currents [98,99]. These neurons also show altered neurofilament expression, indicating molecular reprogramming [100]. Classical studies by de Groat, Yoshimura, and colleagues demonstrated that such changes make bladder afferent neurons more responsive to bladder distension or chemical stimulation, thereby promoting detrusor overactivity and abnormal voiding reflexes [101]. At the molecular level, NGF, PACAP, and other neurotrophic or neuropeptide signals are upregulated, suggesting persistent sensitization of bladder afferent DRG neurons after SCI [102,103]. These animal model studies provide relatively strong mechanistic support for the involvement of intrinsic excitability remodeling of primary sensory neurons in post-SCI bladder dysfunction and suggest that lower urinary tract dysfunction after SCI cannot be explained solely by loss of supraspinal control. However, these specific electrophysiological and molecular changes still lack direct functional validation in humans.

### 5.2. Interstitial Cystitis/Bladder Pain Syndrome

IC/BPS is a chronic syndrome characterized by unpleasant sensations perceived to be related to the bladder, including pain, pressure, or discomfort, often accompanied by frequency and urgency [3,104]. Unlike SCI-related NB, IC/BPS lacks a single, well-defined neural lesion and is heterogeneous with respect to urothelial barrier dysfunction, local inflammation, and peripheral or central sensitization. Nevertheless, sensory neuron-mediated afferent hyperexcitability and neurogenic inflammation are considered important contributors to chronic IC/BPS in at least some phenotypes [3,105].

The peripheral pathological onset of IC/BPS mainly involves urothelial barrier dysfunction and impaired sensory transduction. Classical studies show that urothelial cells derived from patients with IC release more ATP under mechanical stretch in vitro [106]; P2X3 receptor expression is further upregulated under similar conditions, and enhanced extracellular ATP signaling may form an autocrine amplification loop [107]. Other contributors may include TRP channels, NGF, and mast cell mediators. Clinical specimens show upregulation of TRPV1, TRPV4, tryptase, and other sensory and inflammatory molecules in the urothelium and submucosa of patients with IC/BPS, with TRPV1 correlated with symptom severity [108]. Animal and mechanistic studies further suggest that NGF overexpression can induce sensory fiber sprouting, upregulation of TRPV1/P2X3, and C-fiber hyperexcitability [109,110]. Together, these mechanisms may contribute to the peripheral sensitization network in IC/BPS. Mast cell activation and release of histamine, tryptase, NGF, and other mediators further disturb epithelial homeostasis and enhance afferent input [111]. Overall, human tissue studies support urothelial, sensory receptor, and immune-related abnormalities in a subset of patients with IC/BPS, whereas animal models further suggest that NGF and mast cell mediators can causally enhance sensory afferent input. Thus, epithelial abnormalities, immune activation, and sensory terminal sensitization may form an interacting peripheral network in some patients, but the causal contribution and dominant role of individual pathways in human disease may vary by phenotype.

Animal models provide relatively direct mechanistic evidence for DRG and spinal sensitization associated with IC/BPS-like pain. In cystitis models, bladder-innervating DRG neurons undergo functional remodeling and become hyperexcitable [112]. Animal studies show increased ERK1/2 phosphorylation and NK1 receptor upregulation in the spinal dorsal horn, suggesting sustained amplification of bladder nociceptive information [113,114]. In cyclophosphamide models, increased BDNF-TrkB signaling is accompanied by astrocyte and microglial activation, inflammatory mediator production, and mechanical hypersensitivity; TrkB blockade or downregulation of BDNF-TrkB signaling reduces pain-like behavior and bladder dysfunction, providing relatively strong preclinical causal support for the involvement of spinal neuroinflammation and neurotrophic factor signaling in the maintenance of IC/BPS-like pain [115,116]. Combined with studies of segmental hyperalgesia in patients with IC/BPS and related reviews, these findings support the view that sensitization from the periphery to the spinal cord and higher centers is an important mechanistic basis for chronic pain, persistent frequency/urgency, and pelvic cross-sensitization in IC/BPS [117], but these findings do not establish that peripheral bladder abnormalities necessarily lead to central sensitization through a fixed sequential pathway from the DRG to the spinal cord and brain.

Sex differences and psychological comorbidities may further contribute to the clinical heterogeneity and sensory phenotypes of IC/BPS. A recent MAPP Research Network analysis of 961 patients with urologic chronic pelvic pain syndrome (UCPPS), including patients with IC/BPS, showed that, although men and women had broadly similar patterns of core urologic symptoms, women exhibited more widespread non-pelvic pain, greater pelvic floor tenderness, and higher self-reported sensory sensitivity, suggesting that sex may modify the extent of generalized sensory amplification rather than define a single disease mechanism [118]. Consistent with this observation, a mixed-methods study specifically examining patients with IC/BPS found that women reported greater pain intensity and a wider distribution of pain than men, whereas substantial psychological distress was observed in both sexes, suggesting potential sex-related differences in pain experience and supportive care needs [119]. The relationship between psychological factors and IC/BPS symptoms also appears to be complex rather than unidirectional. In a prospective MAPP study, greater widespread pain, non-urological symptom burden, and poorer general health were associated with less favorable longitudinal outcomes, while psychosocial factors such as perceived stress and pain catastrophizing were also associated with pain trajectories [120]. Moreover, in a prospective study of patients with IC/BPS, baseline anxiety severity did not predict treatment response, although improvement in bladder symptoms was accompanied by improvement in anxiety, further arguing against a simple model in which psychological distress acts as a unidirectional cause of bladder dysfunction [121]. Taken together, current evidence supports considering sex, psychological distress, and widespread pain as clinically relevant modifiers of sensory processing, disease burden, and potentially treatment response in IC/BPS, rather than as established independent causal drivers. Future studies and clinical trials may therefore benefit from prespecified stratification according to sex, pain distribution, pelvic floor tenderness, and measures of anxiety, depression, perceived stress, and pain catastrophizing to better distinguish patients with predominantly bladder-localized abnormalities from those with more generalized sensory amplification.

**Figure 5:**
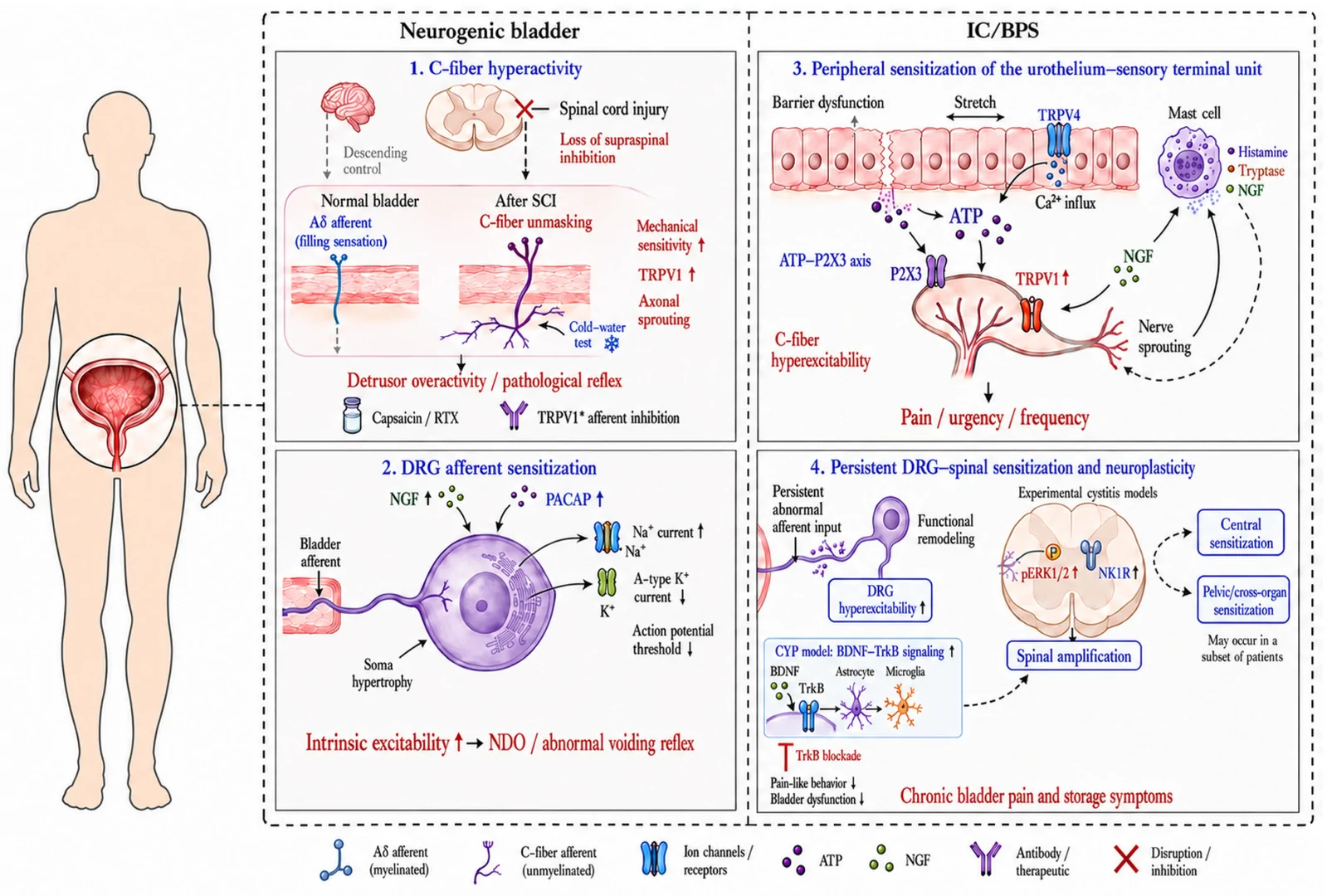
Major mechanisms of sensory neuron pathological remodeling in NB and IC/BPS. Left: SCI-related neurogenic bladder. Human functional and tissue studies support recruitment of C-fiber-related reflexes, TRPV1-related tissue changes, and some clinical effects of capsaicin/RTX desensitization; the specific mechanisms shown in the figure, including C-fiber axonal sprouting and mechanical sensitization, as well as DRG neuronal somatic hypertrophy, altered Na+/K+ currents, and reduced excitability thresholds, are derived mainly from animal SCI models. Right: IC/BPS. Human tissue and ex vivo studies support abnormal urothelial ATP release, P2X3/TRPV1-related changes, and selected immune cell abnormalities; NGF-induced neurite sprouting, C-fiber hyperexcitability, and persistent DRG/spinal sensitization involving ERK1/2, NK1R, and related pathways are supported mainly by animal cystitis models.

Overall, sensory neuron involvement in IC/BPS may begin with peripheral sensitization driven by urothelial ATP, TRP channels, and NGF and become consolidated through persistent DRG/spinal sensitization and neural plasticity, ultimately producing chronic bladder pain and storage symptoms (Figure 5). Sex, age, hormonal status, and the microbiota may modify the urothelial barrier, immune responses, and sensory excitability, thereby contributing to IC/BPS heterogeneity. Current human evidence for these modifiers is mainly observational, however, and causality remains unestablished; they are therefore better viewed as potential stratification variables than as confirmed disease mechanisms.

## 6. Therapeutic Strategies Targeting Bladder Sensory Pathways and the Neuro-Immune Axis

From a translational perspective, interventions directed at bladder sensory pathways do not occupy the same evidentiary tier. BoNT-A, beta3-adrenoceptor agonists, SNM, and PTNS/TTNS have comparatively substantial clinical use or clinical study foundations. Intravesical capsaicin or resiniferatoxin desensitization has some human support in SCI-related NDO, but its durability, repeat treatment requirements, and current clinical role remain uncertain; randomized IC/BPS trials have been inconsistent. TRPV4, TRPA1, CaV3.2, Nav1.8/Nav1.9, ASIC3, P2X3, and several neuroimmune pathways are supported mainly by animal or ex vivo mechanistic studies. Accordingly, this section distinguishes (1) clinically used or clinically validated approaches, (2) approaches with human evidence but uncertain efficacy, and (3) targets supported predominantly by preclinical evidence, rather than presenting them as equally mature therapies.

### 6.1. Pharmacological and Molecular Interventions

**TRP-channel targeting**

Preclinical evidence indicates that TRPV1 contributes to inflammation-associated afferent sensitization. Sustained chemogenetic inhibition of TRPV1-positive bladder afferents improves voiding efficiency, reduces hyperreflexia, and limits abnormal post-injury sprouting in mice [122]. TRPV1 deletion suppresses cystitis-induced bladder and hindpaw mechanical hypersensitivity without preventing local inflammation or inflammatory gene induction, suggesting a predominant role in sensory sensitization rather than initiation of inflammation [123]. In rodents, selective pharmacological blockade raises the micturition reflex threshold and suppresses capsaicin- or citric acid-evoked reflex facilitation [124]; the antagonists GRC-6211 and JTS-653 likewise reduce pathological afferent input or bladder overactivity in experimental models [125,126]. These findings establish biological plausibility, but they do not constitute clinical efficacy evidence. Human interventional evidence concerns agonist-induced desensitization rather than selective TRPV1 antagonism. Intravesical resiniferatoxin (RTX) or capsaicin can reduce detrusor hyperreflexia, increase bladder capacity, and improve incontinence in some patients with neurogenic bladder, but the duration of benefit, need for repeated treatment, and long-term safety remain uncertain; RTX is generally better tolerated than capsaicin [127]. However, a large randomized, double-blind, placebo-controlled trial in IC/BPS did not show overall superiority of RTX over placebo [128]. Potential explanations include marked IC/BPS heterogeneity, pain mechanisms not dominated by TRPV1-positive C fibers, established central sensitization, difficulty balancing exposure with tolerability, and a substantial placebo response. Therefore, there is currently no high-quality contemporary evidence supporting routine TRPV1 agonist desensitization in IC/BPS; if this strategy retains clinical research value, it is more appropriately limited to highly selected, refractory NDO phenotypes. In addition, repeated or overly profound desensitization may cause persistent deafferentation and potentially impair protective bladder sensation; its reversibility and long-term clinical consequences remain inadequately defined. TRPV4 is another important TRP-family channel in urothelial mechanochemical transduction, but the corresponding therapeutic evidence remains predominantly preclinical. In rat cystitis or bladder hypersensitivity models, TRPV4 blockade reduces ATP release, frequency, and pelvic sensitivity [21,129]. In mice, acute LPS stimulation can also induce rapid frequency through urothelial TRPV4 and regulate early inflammatory responses, linking innate immunity to sensory transduction [130]. TRPA1 also contributes to inflammatory bladder hypersensitivity. In LPS- or CYP-induced models, TRPA1 deletion or blockade reduces frequency, bladder pain-like behavior, and mechanical hypersensitivity, with relatively limited effects on basal voiding and local inflammation [131]. Overall, these experimental findings support a role for TRP channels in pathological sensory transduction, but high-quality interventional evidence confirming the efficacy and safety of TRPV4- or TRPA1-targeted treatment in human NB or IC/BPS is lacking. Selective TRPV4/TRPA1 targeting should therefore still be positioned as a preclinical candidate strategy.

**Opioid receptor**

Opioid signaling can modulate abnormal bladder afferent input and pathological micturition reflexes, but much of the mechanistic evidence remains preclinical. In inflammatory bladder pain, mu, delta, and mu/delta heteromeric receptors are upregulated in bladder-related DRG neurons, and peripheral dual-function ligands reduce mechanotransduction in bladder distension-sensitive neurons and alleviate pain [132]. Immune-mediated endogenous opioid analgesia may also operate in the bladder, as T lymphocytes recruited to the mucosa can exert antinociceptive effects through peripheral opioid receptors [133]. Peripheral kappa opioid receptor agonists can inhibit mechanosensitive afferent responses to bladder distension, whereas central mu receptor activation may suppress the micturition reflex and cause urinary retention [134,135]. Translationally, promising approaches include local or peripherally restricted opioid strategies and the FQ/NOP receptor pathway. Intravesical N/OFQ increases bladder capacity, raises the detrusor overactivity threshold, and improves incontinence in patients with SCI-related neurogenic detrusor overactivity [136,137]. Therefore, N/OFQ represents a limited but direct human signal, whereas broader peripherally restricted opioid strategies still require clinical validation and should not have therapeutic value inferred solely from animal analgesic findings.

**Ion channel**

Ion-channel targets provide another strategy for reducing sensory neuron hyperexcitability. CaV3. 2, a T-type calcium channel, is expressed in peripheral nociceptive terminals, DRG, and spinal synapses and contributes to chronic visceral pain [138,139]. In animal studies, selective blockers such as TTA-A2 and ABT-639 suppress bladder distension-evoked afferent responses and nocifensive behavior while having little effect on bladder compliance, suggesting that CaV3. 2 primarily amplifies afferent excitability [140]. Furthermore, the endogenous H2S/CSE pathway can further enhance cystitis-related hypersensitivity by activating CaV3.2, providing a mechanism by which inflammatory signals are converted into DRG hyperexcitability [141]. In parallel, the voltage-gated calcium channel auxiliary subunit α2δ-1, although not a pore-forming subunit, further enhances neuronal excitability by promoting the membrane trafficking and functional expression of high-voltage-activated calcium channels. In cystitis models, blockade of α2δ-1 attenuates bladder hypersensitivity and inflammatory responses, accompanied by reduced NF-κB activity [142]. Moreover, α2δ ligands such as gabapentin and pregabalin have been shown to reduce chronic bladder pain hypersensitivity and improve certain lower urinary tract functions [143].

TTX-resistant sodium channels also contribute to abnormal bladder sensation. Nav1.8 is required for afferent activation after chemical bladder irritation in rats [144], and post-SCI NGF signaling can drive Na+ channel plasticity, increase DRG excitability, and facilitate pathological C-fiber reflexes [99]. Nav1.9 deletion in mice attenuates PGE2-induced afferent sensitization and the reduction in bladder capacity caused by cyclophosphamide, implicating this channel in inflammatory bladder dysfunction [145]. ASIC3 participates in acid-sensitive afferent signaling, and the ASIC blocker A-317567 increases capacity and reduces acid-induced hyperreflexia in animal models [146,147]. Overall, CaV3.2, Nav1.8/Nav1.9, alpha2delta-1, and ASIC3 can modulate bladder-related DRG excitability and pain hypersensitivity phenotypes in animal models, providing an important preclinical mechanistic basis for lowered activation thresholds, high-frequency firing, and persistent sensitization. However, sufficient human functional and interventional evidence is lacking; their pathogenic contribution, efficacy, safety, and patient selection criteria in human NB and IC/BPS remain undefined. These channels should therefore be viewed as candidate targets at an early translational stage (Table 1).

**Anti-inflammatory agents and neuromodulators**

Combined anti-inflammatory and neuromodulatory strategies targeting sensory neurons are reshaping the therapeutic framework for bladder disorders, particularly IC/BPS and neurogenic bladder. Botulinum toxin type A (BoNT-A) has been widely used for refractory neurogenic detrusor overactivity and overactive bladder [148]. Its well-established pharmacological action is to cleave the synaptic protein SNAP-25, thereby inhibiting acetylcholine release from cholinergic nerve terminals and reducing detrusor overactivity [149]. Beyond this classical effect, a single-center, randomized, double-blind controlled trial showed that BoNT-A treatment improved bladder pain and storage symptoms in patients with IC/BPS, accompanied by reductions in bladder tissue or urinary NGF levels among responders [150]. In addition, biopsy studies from other human bladder disorders have shown that BoNT-A can downregulate the suburothelial sensory receptors TRPV1 and P2X3 [151], suggesting that its therapeutic effects may extend beyond detrusor relaxation to modulation of the urothelium–sensory nerve axis and neurogenic inflammation [152]. Animal studies suggest that the HMGB1-RAGE-CSE/H_2_S-CaV3.2 pathway may convert local inflammatory signals into DRG hyperexcitability [153]. Whether this constitutes a key mechanism of anti-inflammatory treatment in human bladder disease has not been validated. In rats, mirabegron has also been shown to inhibit mechanosensitive bladder afferents and microcontractions; the contribution of this sensory mechanism to its clinical efficacy in humans remains uncertain [154].

### 6.2. Neuromodulation

Neuromodulatory therapies for bladder disorders, including sacral neuromodulation (SNM), tibial neuromodulation (PTNS/TTNS), pudendal nerve stimulation (PNS), and electroacupuncture or transcutaneous electrical stimulation, are no longer considered to simply suppress the micturition reflex arc (Table 2). Their effects may involve systematic modulation of the local urothelium–immune–sensory nerve microenvironment and peripheral–central bladder control circuits [155]. SNM, TNS, and electroacupuncture have shown clinical benefits in selected lower urinary tract disorders; however, the evidence supporting their clinical efficacy and that supporting specific molecular mechanisms are not equally mature. Human studies have mainly reported changes in symptom scores, urodynamic outcomes, or functional neuroimaging, whereas alterations in TRP channels, P2X3 receptors, and endogenous opioid signaling have been derived largely from animal models. Therefore, these molecular changes are better regarded as candidate mechanisms rather than mechanisms validated in human NB or IC/BPS.

**SNM**

Clinically, SNM and tibial neuromodulation are established options for refractory overactive bladder and have been extended to selected patients with IC/BPS and NB. A meta-analysis of 17 studies involving 583 patients with refractory IC/BPS reported a pooled treatment success rate of 84% [156]. These approaches improve urinary frequency, urgency, urge incontinence, and some pain-related symptoms [157,158]. In IC/BPS, SNM may also alter urinary inflammatory biomarker profiles. Successful implantation has been associated with reductions in sIL-1ra, MCP-1/CCL2, and CCL5 and downward trends in CXCL-1, CXCL-10, IL-8, VEGF, and PDGF, suggesting modulation of local inflammation and chemokine networks [159]. Functional imaging studies demonstrate that SNM changes urgency-related brain activity in women with OAB and alters brain response patterns in Fowler syndrome, supporting multilevel peripheral–central remodeling [160,161]. Current human mechanistic evidence remains biomarker- or imaging-correlative and cannot establish a causal mechanism for clinical efficacy. Candidate predictors such as age, previous treatment, bladder contractility, and procedural factors have shown inconsistent performance across cohorts; no sufficiently validated clinical or molecular marker currently reliably identifies SNM responders before treatment.

**PTNS**

Mechanistic evidence for PTNS/TTNS is more heavily preclinical. Animal studies of PTNS provide more direct evidence linking nerve stimulation to local inflammation and sensory abnormalities. In post-SCI NB rats, PTNS improves urodynamics, reduces lamina propria inflammation, decreases neutrophil and monocyte infiltration, alleviates edema and vascular congestion, and partly normalizes TRP/P2X3-related molecules in the bladder and DRG [162]. In acid-induced bladder overactivity, the inhibitory effect of tibial nerve stimulation depends on opioid receptors and metabotropic glutamate receptors [163]. Foot stimulation also requires opioid receptors, with different opioid receptor subtypes contributing distinct effects [164,165]. The integrity of the brainstem is necessary for tibial inhibition of the micturition reflex, indicating that PTNS/TTNS operates through somatic afferent–brainstem–spinal reflex circuits rather than peripheral stimulation alone [166]. In a larger retrospective OAB cohort, success according to strict criteria was 29.9% at 8 weeks and 41.3% at 12 weeks; when mild improvement was included, response rates increased to 54.2% and 60.5%, respectively [167]. In a small exploratory IC/BPS study, the response rate at 12 weeks according to the prespecified Global Response Assessment criterion was 30%, although approximately 70% of patients reported some degree of subjective improvement [168]. These data suggest that some patients may benefit, but they do not support IC/BPS and NB as uniform. Clinical predictors proposed in retrospective studies have not been consistently replicated, and there is currently no validated biomarker for routine patient selection. These findings provide a mechanistic basis for the multilevel modulatory effects of PTNS, although whether the same molecular pathways mediate its therapeutic effects in human IC/BPS or NB remains to be validated.

**PNS**

Animal studies indicate that PNS may be particularly relevant for suppressing bladder pain and hypersensitivity. In neonatal cystitis in rats, bilateral PNS suppresses bladder distension-evoked visceromotor responses, and intrathecal blockade of serotonergic, GABAergic, or glycinergic mechanisms attenuates this analgesic effect [169]. In cat models, pudendal inhibition of bladder overactivity involves GABA_A, glycine, NMDA/AMPA, and beta-adrenergic mechanisms [170,171,172]. Sensory pudendal nerve stimulation can increase bladder capacity through sympathetic mechanisms in CYP cystitis models [173]. These animal results suggest that the inhibitory effects of PNS may involve coordinated actions among spinal inhibitory neurotransmission, sympathetic modulation, and sensory afferent gating, but this remains predominantly preclinical mechanistic evidence.

**Electroacupuncture**

Electroacupuncture and transcutaneous electrical stimulation are also reported to modulate bladder sensory pathways. In OAB rats, electroacupuncture downregulates P2X3 expression in peripheral bladder tissues, DRG, and the spinal cord, increasing bladder capacity and suppressing overactivity [174]. In SCI-related detrusor hyperreflexia, electroacupuncture reduces sacral spinal c-fos expression and may suppress C-fiber-driven spinal sensitization [175]. In IC/cystitis models, electroacupuncture decreases TRPV1/P2X3 expression and reduces IL-6, TNF-alpha, mast cell tryptase, and related inflammatory mediators [176,177,178]. These findings indicate that some neuromodulatory therapies do not merely “silence” neural activity but can also modulate inflammatory responses and directly interfere with the peripheral transduction of aberrant sensory signals. In acute urinary retention-related models and limited clinical studies, electroacupuncture reduces urinary ATP levels and downregulates TRPV1 expression, improving intravesical pressure and abnormal voiding patterns through the TRPV1/ATP pathway [179]. These animal and limited clinical studies suggest that transcutaneous stimulation and electroacupuncture may, beyond affecting the micturition reflex, be accompanied by changes in inflammatory mediators, ATP, and sensory-related signals such as TRPV1/P2X3. However, it remains uncertain whether these molecular changes directly mediate their clinical effects; they are better considered candidate mechanisms requiring further human validation.

## 7. Conclusions and Perspectives

Sensory neurons integrate bladder stretch, chemical irritation, and microbe-associated signals, thereby contributing to coordination of the micturition reflex, local immunity, and pain perception. Under physiological conditions, their interactions with the urothelium and immune cells support bladder homeostasis. In disease, abnormal mediator release, afferent sensitization, and plasticity across DRG–spinal–brain networks may contribute to symptoms of NB and IC/BPS. Importantly, SCI-related NB has a comparatively well-defined pattern of neural circuit reorganization, whereas the etiology and clinical phenotypes of IC/BPS are substantially more heterogeneous; shared sensory amplification pathways should not be interpreted as identical disease mechanisms.

Major limitations remain. The functions of specific bladder sensory neuron subpopulations are incompletely resolved, and many molecular mechanisms are supported primarily by cyclophosphamide cystitis, SCI, NGF overexpression, or UPEC infection models, with less direct human tissue or functional evidence. A sensory neuron-centered framework also does not imply that afferents act alone. Sympathetic and parasympathetic pathways regulate detrusor activity, local blood flow, and immune responses, whereas interstitial cells and myofibroblasts contribute to mechanical signaling and tissue remodeling. Sex, age, hormonal state, microbiota, and psychological comorbidity may further modify disease phenotypes. These factors fall outside the principal scope of this review but represent important interfaces with sensory pathways for future investigation.

Future work should move from cataloguing candidate mechanisms to validating patient stratification. Single-cell and spatial profiling of human bladder biopsies, urinary proteomics and metabolomics, barrier and neuroimmune biomarkers, urodynamics, sensory testing, and neuroimaging should be integrated to link tissue changes, neural dysfunction, clinical phenotypes, and treatment response. Similar multimodal biomarker strategies have shown potential to improve diagnostic performance and risk stratification in other bladder diseases; for example, combining a urinary molecular assay with conventional cytology improved the diagnostic performance of urothelial lesion surveillance [180]. Such findings support the broader concept that integrating complementary biomarker modalities with clinical phenotyping may enhance precision stratification in bladder disorders. BoNT-A, beta3-adrenoceptor agonists, SNM, and PTNS/TTNS have a clinical foundation, but predictors of response and long-term benefit require definition. TRPV1 desensitization has some support in SCI-related NDO but should not be generalized to all IC/BPS cases; P2X3, TRPV4, TRPA1, CaV3.2, Nav1.8/Nav1.9, ASIC3, CGRP, and HMGB1 remain candidate targets. Mechanism-enriched stratified trials that integrate animal, human tissue, and clinical response evidence will be essential for translating sensory neuron biology into predictable precision therapy.

## Figures and Tables

**Table 1:** Pharmacological and molecular strategies targeting bladder sensory and neuroimmune pathways.

Target/Pathway	Representative Intervention	Core Mechanism	Reported Bladder Effects	Evidence Context	References
TRPV1	RTX or capsaicin; selective antagonists; genetic/pharmacological inhibition	Desensitizes C fibers, raises the micturition reflex threshold, and suppresses afferent firing and sensory sensitization	Reduces detrusor hyperreflexia, bladder overactivity, urinary urgency/frequency, and pain-related hypersensitivity	Preclinical and clinical evidence; sensory inhibition may occur without suppressing local inflammatory gene expression	[122,123,124,125,126,127,128]
TRPV4	TRPV4 blockers or other targeted inhibition	Reduces Ca^2+^ influx and urothelial ATP release; modulates early LPS-associated responses	Reduces voiding frequency, afferent amplification, and pelvic hypersensitivity	Predominantly preclinical mechanistic evidence	[21,129,130]
TRPA1	Pharmacological blockade	Suppresses LPS- or cyclophosphamide-associated inflammatory sensory signaling	Reduces urinary frequency, bladder pain-like behavior, and mechanical hypersensitivity	Preclinical evidence	[131,132]
Opioid and NOP signaling	Bifunctional opioid ligands; nociceptin/orphanin FQ	Engages mu/delta heteromers or peripheral opioid pathways to inhibit nociceptive mechanotransduction	Attenuates distension-sensitive afferent activity and bladder pain	Preclinical and exploratory clinical evidence	[133,134,135,136]
Calcium-channel pathways (CaV3. 2/α2δ-1)	TTA-A2; ABT-639; H_2_S/CSE inhibition; gabapentin; pregabalin	Suppresses CaV3. 2-dependent afferent responses, calcium-channel-associated excitability, and NF-κB signaling	Reduces distension-related nociception, bladder inflammation, hypersensitivity, and chronic pain sensitization	Preclinical bladder evidence; interventions are not bladder-specific	[138,139,140,141,142,143]
Nav1. 8/Nav1. 9	Experimental genetic or pharmacological inhibition	Limits NGF- or PGE_2_-associated sodium current plasticity and afferent hyperexcitability	May reduce C-fiber-dominated abnormal reflexes, inflammatory bladder dysfunction, and pain	Experimental/preclinical evidence; selective clinical therapies remain unavailable	[99,144,145]
ASICs/ASIC3	A-317567	Inhibits acid-sensitive signaling in bladder sensory neurons	Increases bladder capacity and reduces acid-induced bladder hyperreflexia	Preclinical evidence	[24,146,147]
BoNT-A	Intradetrusor botulinum toxin A injection	Downregulates bladder NGF and P2X3/TRPV1 expression in suburothelial sensory fibers	Improves pain, urgency, and bladder capacity in IC/BPS	Clinical and translational evidence; treatment response varies among phenotypes	[148,149,150,151,152]
HMGB1-RAGE-CSE/H_2_S-CaV3. 2	HMGB1/RAGE blockade	Interrupts macrophage-derived HMGB1 signaling to the CSE/H_2_S-CaV3. 2 pain pathway	Reduces bladder inflammation, bladder pain, and referred hyperalgesia	Preclinical evidence; no established clinical validation	[153]

Note: Evidence context reflects the highest and predominant level of evidence supporting each therapeutic strategy. “Preclinical evidence” refers primarily to animal or in vitro mechanistic studies; “human tissue/ex vivo evidence” refers to findings from human bladder tissue or isolated human specimens; “clinical evidence” refers to studies involving patients, including observational, interventional, and randomized controlled studies where available. When both preclinical and clinical evidence are listed, the mechanistic basis and therapeutic efficacy may be derived from different levels of evidence and should not be interpreted as equally mature.

**Table 2:** Neuromodulatory strategies targeting bladder sensory and neuroimmune pathways.

Modality	Principal Pathways	Core Mechanism	Reported Effects	Evidence Context	References
Sacral neuromodulation (SNM)	Sacral afferent/efferent circuits; inflammatory mediators; central bladder networks	Modulates peripheral–central bladder control, urinary chemokine profiles, and urgency-related brain activity	Improves frequency, urgency, urgency urinary incontinence, and selected pain-related symptoms	Human clinical and mechanistic studies	[156,157,158,159,160,161]
Tibial nerve stimulation (PTNS/TTNS)	TRP/P2X3 signaling; opioid and metabotropic glutamate receptors; brainstem–spinal reflexes	Reduces local inflammatory signaling and sensory sensitization while engaging central–peripheral inhibitory networks	Improves urodynamic parameters and suppresses bladder overactivity and abnormal afferent activity	Mechanistic evidence in the cited studies is predominantly preclinical	[162,163,164,165,166,167,168]
Pudendal nerve stimulation (PNS)	Spinal 5-HT, GABA, glycine, glutamate, sympathetic, and β-adrenergic pathways	Enhances spinal inhibitory neurotransmission, sensory afferent gating, and autonomic regulation	Increases bladder capacity and reduces bladder overactivity, hypersensitivity, and visceral pain	Preclinical animal evidence	[169,170,171,172,173]
Electroacupuncture	P2X3, TRPV1, urothelial ATP, inflammatory mediators, and spinal c-Fos	Suppresses peripheral and spinal sensory signaling and reduces inflammatory-mediator expression	Improves bladder capacity, intravesical pressure, voiding patterns, and pain-related behavior	Primarily preclinical evidence, with a limited clinical component in the cited literature	[174,175,176,177,178,179]

## Data Availability

No new data were created or analyzed in this study. Data sharing is not applicable to this article.

## Abbreviations

The following abbreviations are used in this manuscript:

5-HT	5-Hydroxytryptamine
ASIC	Acid-sensing ion channel
ATP	Adenosine triphosphate
BoNT-A	Botulinum neurotoxin type A
CALCRL	Calcitonin receptor-like receptor
CCL	C-C motif chemokine ligand
CGRP	Calcitonin gene-related peptide
CSE	Cystathionine γ-lyase
CYP	Cyclophosphamide
DRG	Dorsal root ganglion
GABA	γ-Aminobutyric acid
H_2_S	Hydrogen sulfide
HMGB1	High-mobility group box 1
IC/BPS	Interstitial cystitis/bladder pain syndrome
ICAM-1	Intercellular adhesion molecule 1
IL	Interleukin
ILC2	Type 2 innate lymphoid cell
LPS	Lipopolysaccharide
MCP-1	Monocyte chemoattractant protein 1
NB	Neurogenic bladder
NDO	Neurogenic detrusor overactivity
NF-κB	Nuclear factor kappa B
NGF	Nerve growth factor
NKA	Neurokinin A
NKB	Neurokinin B
NK1R	Neurokinin 1 receptor
NK2R	Neurokinin 2 receptor
NK3R	Neurokinin 3 receptor
N/OFQ	Nociceptin/orphanin FQ
NOP	Nociceptin/orphanin FQ receptor
OAB	Overactive bladder
P2X3	Purinergic receptor P2X3
PACAP	Pituitary adenylate cyclase-activating polypeptide
PAG	Periaqueductal gray
PDGF	Platelet-derived growth factor
PDGFR	Platelet-derived growth factor receptor
PGE_2_	Prostaglandin E_2_
PGP9. 5	Protein gene product 9. 5
PNS	Pudendal nerve stimulation
PTNS	Percutaneous tibial nerve stimulation
RAGE	Receptor for advanced glycation end products
RAMP1	Receptor activity-modifying protein 1
ROS	Reactive oxygen species
RTX	Resiniferatoxin
SCI	Spinal cord injury
SNM	Sacral neuromodulation
SP	Substance P
TLR4	Toll-like receptor 4
TNF-α	Tumor necrosis factor alpha
TRP	Transient receptor potential
TRPA1	Transient receptor potential ankyrin 1
TRPM3	Transient receptor potential melastatin 3
TRPM8	Transient receptor potential melastatin 8
TRPV1	Transient receptor potential vanilloid 1
TRPV4	Transient receptor potential vanilloid 4
TTNS	Transcutaneous tibial nerve stimulation
TTX	Tetrodotoxin
UPEC	Uropathogenic *Escherichia coli*
VEGF	Vascular endothelial growth factor
VIP	Vasoactive intestinal peptide
VPAC1	Vasoactive intestinal peptide receptor 1
VPAC2	Vasoactive intestinal peptide receptor 2
